# Continual learning approaches for single cell RNA sequencing data

**DOI:** 10.1038/s41598-023-42482-7

**Published:** 2023-09-15

**Authors:** Gorkem Saygili, Busra OzgodeYigin

**Affiliations:** Cognitive Sciences and Artificial Intelligence, Tilburg School of Humanities and Digital Sciences, Tilburg University, Warandelaan 2, 5037 AB Tilburg, The Netherlands

**Keywords:** Computational biology and bioinformatics, Machine learning

## Abstract

Single-cell RNA sequencing data is among the most interesting and impactful data of today and the sizes of the available datasets are increasing drastically. There is a substantial need for learning from large datasets, causing nontrivial challenges, especially in hardware. Loading even a single dataset into the memory of an ordinary, off-the-shelf computer can be infeasible, and using computing servers might not always be an option. This paper presents continual learning as a solution to such hardware bottlenecks. The findings of cell-type classification demonstrate that XGBoost and Catboost algorithms, when implemented in a continual learning framework, exhibit superior performance compared to the best-performing static classifier. We achieved up to 10% higher median F1 scores than the state-of-the-art on the most challenging datasets. On the other hand, these algorithms can suffer from variations in data characteristics across diverse datasets, pointing out indications of the catastrophic forgetting problem.

## Introduction

Two significant technological breakthroughs, namely machine learning and single-cell RNA sequencing (scRNA-seq), have revolutionized our contemporary era. The remarkable progress in these fields paved the way to even more remarkable accomplishments through the integration of machine learning algorithms in the analysis of scRNA-seq data^1^. The considerable growth of the number of studies in this domain has led to datasets of thousands of samples and hundreds of cell populations^2–12^. Although there are inspiring studies on creating benchmark scRNA-seq datasets^13^, as emphasized in^14^, there is not yet a single reference atlas containing all the cell types. Hence, learning from many datasets together is an essential task, and there have been recent efforts to build up classifiers that enable this, such as scHPL^14^ and treeArches^15^.

Machine learning algorithms have been used for the classification of cell types using scRNA-seq data for over a decade. One of the most extensive benchmark articles on this topic compared the performances of 22 classification algorithms on 27 publicly available datasets^13^. These machine learning algorithms consist of simple classifiers, such as K-Nearest Neighbors (KNN)^16,17^, and more complex ones build upon neural networks^18,19^. According to their results, linear support vector machine (SVM) classifier was identified as the top performer. Furthermore, they measured the complexity of the datasets based on the variation in performance between the classifiers. They also measured the complexities of the datasets, and based on their outcomes, large-sized datasets such as Zheng 68K^10^ and AMB^9^ were among the most challenging for the classifiers to classify the cell types. In addition to their challenges in classification, loading these datasets into the memory of an ordinary off-the-shelf computer is also a challenge due to their demand for memory. This non-trivial challenge can be addressed via continual learning (CL) algorithms, as such algorithms are capable of learning from streams of data without the need to use all of it at once for training.

There are a variety of terminologies for CL, such as lifelong learning^20–23^, incremental learning^24–26^, and sequential learning^27^. Due to the existence of a variety of terminologies, what exactly CL refers to has been largely debatable. In a recent survey of van den Ven et al.^28^, CL was categorized into three distinct classes: task-incremental, domain-incremental, and class-incremental. Task-incremental refers to CL algorithms that aim to learn incrementally between various different tasks. Domain-incremental learning algorithms, on the other hand, focus on the same task over different distributions of data, whereas class-incremental algorithms continually learn the same task on batches with varying numbers of classes. De Lange et al.^29^ surveyed the task-incremental domain, stating that CL aims to extend its knowledge through a stream of data. Similarly, Lesort et al.^30^ defined the CL as learning from a stream of data that is not available as a whole at once. Hence, it should not be possible to reuse the same training data repetitively for training the CL classifier. We adopted this definition for CL and argue that a classifier that learns through batches of training data without considering the same batch twice is a CL approach. This strategy of learning through batches of training data rather than the whole of it at once enables the utilization of smaller resources in terms of memory, providing a solution to the before-mentioned hardware challenge. In addition to its advantage in terms of memory, CL algorithms also facilitate the development of more generalizable models without compromising data privacy by utilizing raw data from multiple institutions without the need to share them explicitly, thus making contributions to preserving data privacy^31^. Not all CL algorithms provide similar performances across the stream of data because of the fundamental trade-off, known as plasticity and stability^32^. Plasticity refers to learning from new data, whereas stability refers to retaining knowledge from previous learning experiences. Understanding how different CL classifiers perform for the classification of cell types using scRNA-seq data is crucial, which constitutes one of the main aspects of this study.

Catastrophic forgetting was introduced by McCloskey and Cohen^33^ towards the end of the 1980s and persists as an important concern for various CL tasks. Catastrophic forgetting, in a nutshell, can be described as the tendency of a machine learning algorithm to forget previously learned information when it is trained on new data. In fact, catastrophic forgetting happens when a CL algorithm has high plasticity but low stability. While harnessing information from larger datasets offers notable advantages, it also entails the inherent risk of potential knowledge loss during the retraining process. This problem has been addressed recently via a variety of different CL strategies^20,23–26,29^.

In this study, we opt to find a solution to learning from large and challenging datasets. To achieve this, we utilized CL algorithms, which enable learning from small subsets of the data continuously rather than from the whole set in one go. Following the common nomenclature, these subsets of the data will be referred to as “batches” throughout the paper.

We outline the contributions of our work as follows:

- We provide a solution to learning from large datasets without the need to load the full dataset into memory while achieving high accuracy on par with the linear SVM and even outperforming on the most complex Zheng 68K dataset.
- Our experimental results update the existing literature by comparing different CL algorithm performances on the most recent scRNA-seq benchmark dataset.
- We demonstrate the performance differences between inter and intra-dataset experiments in the CL approach in terms of catastrophic forgetting.

## Results

### scRNA-seq benchmark results with CL algorithms signifies XGBoost and CatBoost as the top performers

**Figure 1:**
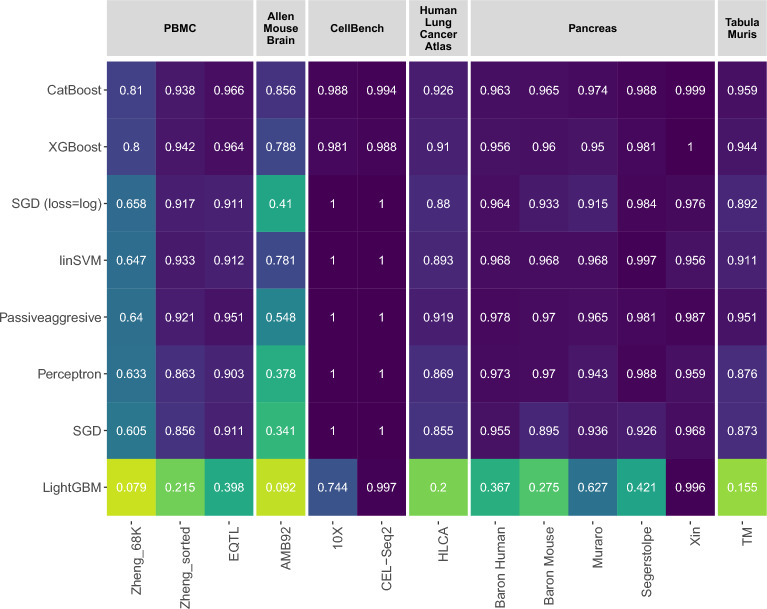
Comparison of intra-dataset experiment results for cell identification. Each number indicates the median F1-score of 5-fold cross-validation for seven different classifiers on each dataset.

In the context of intra-dataset evaluation, we conducted a comprehensive assessment of the performance of six different CL classifiers, along with a linear support vector machine (SVM), across a total of 13 datasets. Each dataset was partitioned into batches using a stratified 5-fold cross-validation approach, and the classifiers were trained using their default parameter settings. Figure 1 presents the median F1 scores. Interestingly, both the XGBoost and CatBoost algorithms exhibited superior performance compared to the linear SVM classifier. It is worth noting that when the loss parameter of the SGD classifier is hinge loss (SGD), it corresponds to the linear SVM. Since it is the default loss function for SGD, it is denoted as SGD in the results. Additionally, when the loss function log_loss is selected, the SGD classifier represents the logistic regression classification algorithm which corresponds to ’SGD (loss=log)’. The outcomes of the evaluation demonstrate the superiority of the CatBoost and XGBoost algorithms over both the static linear SVM and linear SVM classifiers within the continual learning (CL) framework. Passive-Aggressive classifier, specifically designed for online learning tasks, surpassed linear SVM on several datasets. All remaining CL classifiers except the LightGBM showed decent performance, if not better than the linear SVM. A more extensive comparison involving models employing different regularization techniques, such as L1, L2, and elasticnet, can be found in Fig. 1 in the Appendix. These results confirm the strong performance of CL algorithms, especially on challenging datasets such as Allen Mouse Brain and Zheng 68K. However, it is important to note that all the batches were from the same dataset for the intra-dataset experiment, causing consecutive batches to be alike.

### Experimental results on the latent space confirm the results of the intra-dataset experiment

**Figure 2:**
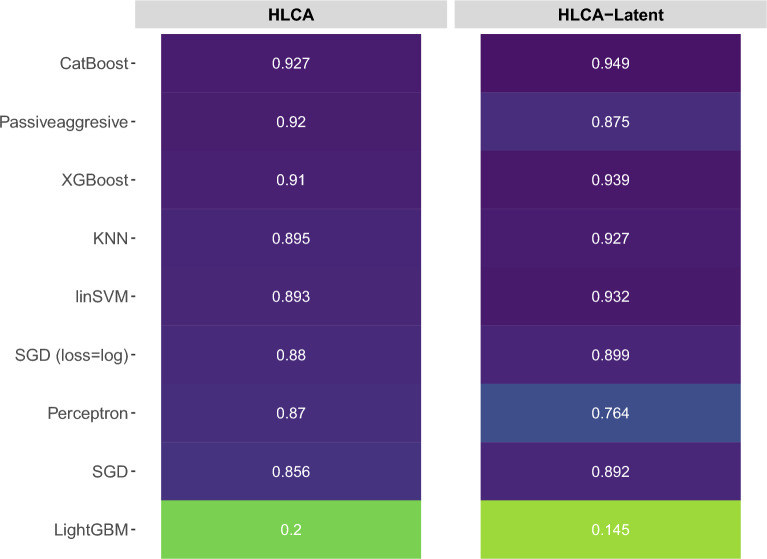
The median F1-scores of all classifiers, including KNN, on high-dimensional and latent spaces of the HLCA dataset from^15^.

scArches^34^ and its recent advanced version with scHPL, treeArches^15^, map the high-dimensional scRNA-seq data to a latent space to combine multiple atlases or datasets for downstream analysis. Although linear SVM had provided top performance for the classification of high-dimensional data, Michielsen et al.^15^ noted in their study that data in the latent space is not as linearly separable as the high-dimensional space, leading them to utilize a KNN classifier rather than linear SVM in their experiments. In this experiment, we also included KNN and used the latent spaces from^15^ to match the classification performances of CL algorithms against linear SVM and KNN on the recently released Human Lung Cell Atlas (HLCA)^34^ dataset. Median F1-scores of all classifiers on the high-dimensional HLCA dataset and its latent space from^15^ can be seen in Fig. 2. Following the previous results, CatBoost was the top performer, and XGBoost provided on-par performance with CatBoost on both high-dimensional data and the latent space. Although the Passive-Aggressive algorithm outperformed linear SVM and KNN on the high-dimensional data, it showed worse performance than the other two in the latent space. In contrast to the outcome of Michielsen et al.^15^, linear SVM and KNN performed on par with each other on high-dimensional and latent spaces for the HLCA dataset. Similar to the previous results, LightGBM was the worst performer in high-dimensional and latent spaces.

### Training over diverse datasets can have a detrimental effect on continual learning performance

**Figure 3:**
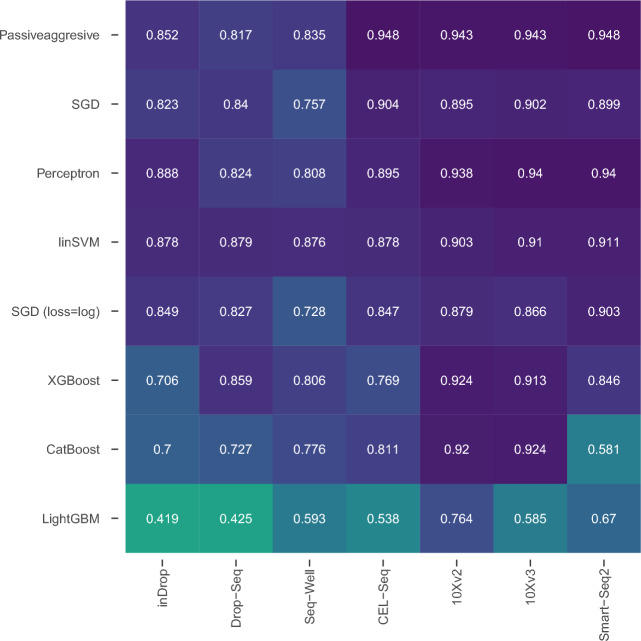
Median F1-scores of inter-dataset experiments. The columns represent the utilization order of the datasets through the training of each classifier. The sorting is done based on the mean of the median F1-scores over all datasets.

One of the major problems encountered in CL is catastrophic forgetting. This phenomenon arises when the consecutive batches used for training exhibit substantial variations, such as those stemming from different populations or datasets. We conducted inter-dataset experiments to find out whether the CL algorithms are affected by catastrophic forgetting. Rather than a set of samples from the same dataset, we trained the classifiers continually with different datasets as batches. The feeding order of datasets into the classifiers is presented in Fig. 3, and the results were sorted from top to bottom based on the mean of the median F1-scores in decreasing order. In this experiment, the top-performing classifier was the Passive-Aggressive classifier, followed by the SGD and the Perceptron. Interestingly, the XGBoost and CatBoost classifiers exhibited suboptimal performance, as they underperformed not only the linear SVM classifier but also all other CL classifiers, with the exception of the LightGBM. A comprehensive comparison of inter-dataset experiments, featuring models employing diverse regularization techniques including L1, L2, and elastic net, is available in Fig. 1 in the Appendix.

**Figure 4:**
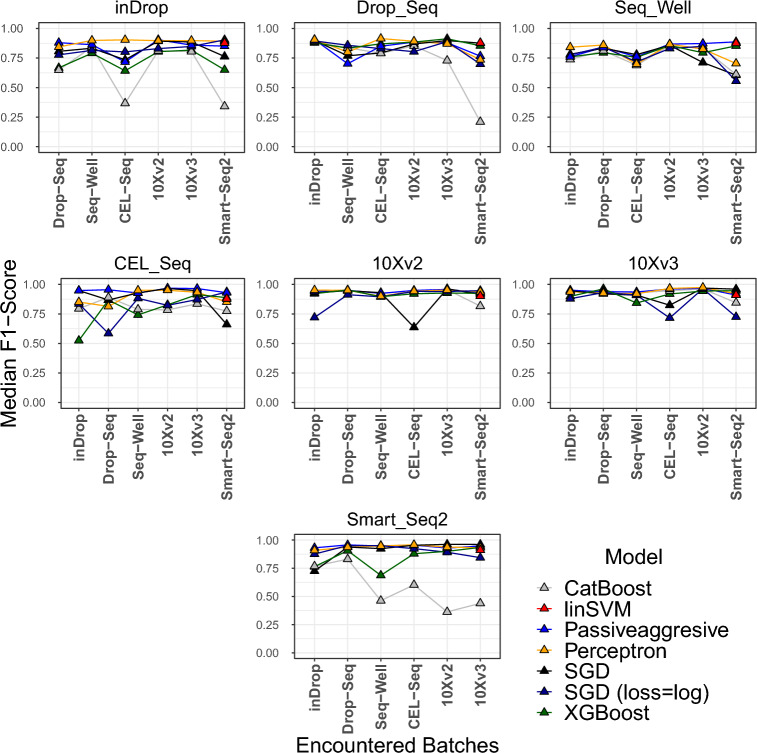
Performance change of continual learning algorithms through batches of datasets for inter-dataset experiments. In each graph, a different dataset was evaluated as the test set, which is described at the top of each graph. The batch order remains the same for all experiments.

Figure 4 displays graphical representations that depict the incremental median F1 scores for each batch, where a distinct dataset is utilized as the test set (specified on top of each graph) for every training batch. These graphs provide valuable insights into the comparative analysis among models on a batch-by-batch basis. Notably, the graphs vividly illustrate the notable influence of the similarity between the training set employed in each batch and the test set on the overall performance of the model. This phenomenon is indicative of catastrophic forgetting. Based on the empirical findings, our analysis suggests that the XGBoost and CatBoost algorithms in the CL setup are susceptible to the catastrophic forgetting phenomenon. In contrast to these two classifiers, which used their last model as the initial model of their next batch, the SGD, Perceptron, and Passive-Aggressive CL algorithms improved their models incrementally from their last state. Eventually, this incremental “fine-tuning” approach adapted to varying distributions between different datasets better than the initialization approach.

### Batch sizes affect the performance of the continual learning algorithms

**Figure 5:**
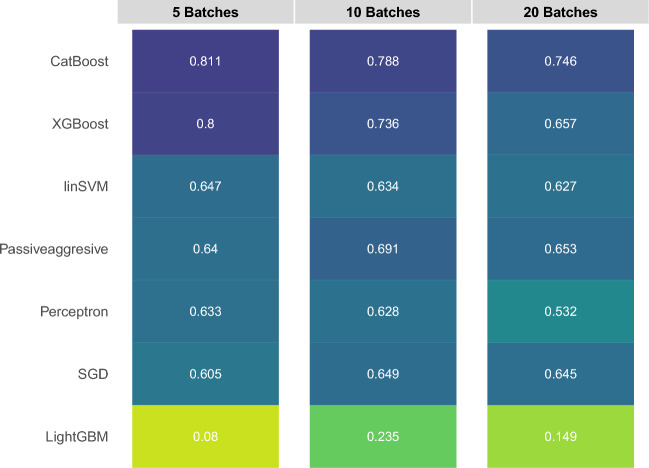
Median F1 scores for stratified batch sizes of 5, 10, and 20 on the Zheng68K dataset.

An important hyperparameter to be decided before applying the CL classifiers is the batch size. The choice of batch size is crucial as having a larger number of smaller-sized batches can introduce diverse distributions that significantly differ from both each other and the overall distribution of the training set. This can potentially result in adaptation difficulties and lower overall performance. To evaluate the impact of batch size, we generated 5, 10, and 20 stratified batches from the highly challenging Zheng 68K dataset. The median F1-scores obtained from each batch are presented in Fig. 5. Similar to the results of the intra-dataset experiment, CatBoost, and XGBoost classifiers were the best performers, outperforming the linear SVM classifier with a large margin. However, their performances were hampered by the batch size, supporting the results of the inter-dataset experiments. Complementary to this, Passive-Aggresive and SGD algorithms, as the top performers of the inter-dataset experiment, were not hampered by the batch size variation as much as the CatBoost and the XGBoost classifiers.

## Discussion

In this study, we compared the performance of seven different classifiers, of which six (CatBoost, XGBoost, LightGBM, Passive-Aggressive, Perceptron, and SGD) were applied in the CL setup. All of the classifiers were trained and tested using a total of 20 datasets for intra- and inter-dataset experiments. One of these datasets, Zheng 68K, was particularly emphasized in the earlier study of Abdelaal et al.^13^ as being the most challenging for all the static classifiers in their comparison. In our intra-dataset experiments, we particularly focused on the performances of our CL classifiers on the Zheng 68K dataset while comparing their performances against the best-performing static linear SVM classifier^13,14^. The results of XGBoost and CatBoost CL classifiers over 5-fold stratified batches outperformed the linear SVM for every dataset, including the Zheng 68K. In addition to XGBoost and CatBoost, SGD and Passive-Aggressive classifiers also showed on-par performance with linear SVM except for the AMB92 (92 cell populations) dataset. It is crucial to highlight that the AMB92 dataset consists of 92 classes with a highly imbalanced distribution among them. When splitting this data into batches, the performance of models in the CL framework can be negatively affected, as some classes could have a notably low number of samples in the batches, even with the application of stratified cross-validation. In all of our experiments, LightGBM in CL setup provided lower performance than the other classifiers. We argue that this low performance is due to the leaf-wise growth strategy of LightGBM^35^ that can produce more complex trees than the other boosting algorithms which, in turn, decreases the plasticity of the algorithm through the batches.

Linearly separable datasets can be classified with high accuracy using the linear SVM classifier. However, not all scRNA-seq datasets are linearly separable. One exception is the latent space of the HLCA dataset. The authors of^15^ used a KNN classifier rather than a linear SVM to address this non-linearity and achieve better classification performance. We tested the CL classifiers against both linear SVM and KNN classifiers on both latent space and high-dimensional versions of the HLCA datasets. Both KNN and linear SVM classifiers showed on-par performances; however, CatBoost, and XGBoost achieved better performances in latent space. This result confirms that CL classifiers have the potential to perform better than static classifiers in latent space and on non-linearly separable datasets. Interestingly, Passive-Aggressive classifier performed better than both KNN and linear SVM on the high-dimensional data; however, its performance got worse in the latent space.

A crucial problem with CL algorithms occurs when the distribution of the data substantially changes along the batches. Such a change particularly occurs when the batches come from different datasets. In these circumstances, the learning algorithm may catastrophically forget what it has learned previously and adapt to the new distribution. In order to analyze the effect of learning through different datasets and to find out whether there is an effect like catastrophic forgetting, we designed inter-dataset experiments using the aligned datasets from^13^. The results showed considerable differences from the intra-dataset experiments. SGD, Perceptron, Passive-Aggressive, the incremental learning classifiers from scikit-learn, outperformed XGBoost and CatBoost substantially while showing better overall performance than linear SVM. It is important to note that XGBoost, CatBoost, and LightGBM are originally static classifiers that were applied in a CL setup. At each batch, they were initialized with their most recent configuration and trained with the new batch afterward. We argue that such a configuration is prone to distributional variations in the batches. The drop in performance through the batches of different datasets can be clearly seen in Fig. 4, especially for the CatBoost classifier. In contrast, Perceptron, SGD, and Passive-Aggressive classifiers incrementally updated their states as they were trained on a new batch. This eventually led to better performance against substantial changes through the batches. Furthermore, it is essential to acknowledge that the order in which batches are presented to the CL algorithms can also have an impact on their performance. This impact is evident in Fig. 4, where the performance of the CL algorithms consistently exhibits a pattern of improvement after training on the 10Xv2 dataset and testing on the 10Xv3 dataset, and vice versa.

An important hyperparameter of our experiments was the number of batches. There are other studies that have also delved into the examination of the role of batch size in various aspects, including one that specifically focused on the buffer size in the replay method of the CL framework^36^. In that study, they investigated the role of buffer size that would be stored and reused in the next batch during training. Interestingly, they found that smaller batch sizes were more effective in mitigating catastrophic forgetting. In our CL approach, contrary to the replay method, we do not reuse any samples for training which is in line with our definition of continual learning. Akin to^13^ we used stratified 5-fold CV to create five batches. To analyze how CL algorithms are affected by the number of re-trainings applied, we utilized 5, 10, and 20 batches and compared the performances. The results showed that CatBoost and XGBoost performed worse as the number of batches increased, i.e., smaller batch sizes. On the other hand, Passive-Aggressive and SGD classifiers were not affected by the increase in the number of batches.

It is important to note that we did not include a deep neural network (DNN) such as a multi-layer perceptron (MLP) in our study. This is mainly because recent state-of-the-art studies achieved considerably higher performance with traditional machine learning algorithms than with MLPs. However, we foresee that better performances can be achieved using continual learning with DNN architectures. In particular, we believe that DNNs can be more successful in the classification of datasets that are not linearly separable. Furthermore, different techniques can also be utilized, such as transfer learning, to carry information from several datasets through pre-trained networks. Yet, the effect of catastrophic forgetting can be a severe factor that limits the use of such techniques, which needs to be researched deeply in a future study.

## Conclusions

We presented an extensive analysis of continual learning classifiers to provide a solution for the training of machine learning algorithms on large scRNA-seq datasets. Intra and inter-dataset experiments, together with experiments on latent space and different numbers of batches showed that continual learning algorithms can provide better classification performances than static best-performing classifiers while being trained on batches of data rather than the entire training set. These results are promising since the size of scRNA-seq datasets have been increasing and learning from multiple datasets has been the primary focus of many recent studies. Although they provide decent performances, some of the continual learning algorithms are hampered more than others when the variation between batches increases substantially.

## Methods

### Classification methods

We evaluated seven classifiers, including a static linear SVM classifier as the baseline. Six of the seven classifiers were configured in a continual setting and gradually trained using batches of data. These six classifiers include XGBoost^37^, CatBoost^38^, LightGBM^35^, and three CL classifiers from scikit-learn library^17^: SGD, Passive-Aggressive, and Perceptron classifiers.

XGBoost, CatBoost, and LightGBM were essentially static classifiers. To train them continually, we utilized initialization. They were first trained using the first batch which was used as the initial configuration for the training with the next batch. Hence, we used the last models of these three classifiers, which were trained using the previous batch as their initial/starting model, to be updated on the recent batch. On the contrary, CL classifiers in scikit-learn ensure CL with their partial_fit function which enables incremental improvements of the model as the new data comes in for training rather than using the last trained model as the initial model of the next batch.

The performances of classifiers depend on the setting of their hyperparameters, and each of them generally has a different number of hyperparameters to be optimized. Furthermore, optimizing these hyperparameters on a dataset may induce overfitting. To be fair to each of these classifiers and to alleviate the risk of overfitting, we used the default values for the hyperparameters of these classifiers akin to^13^. The only exception happened with the SGD classifier for which different loss functions correspond to different classifier types. Stochastic Gradient Descent (SGD) is an optimization method for the model parameters, $\textbf{w}$, while minimizing the objective function, $Q(\textbf{w})$, with an iterative approach:

1 $$\begin{aligned} Q(\textbf{w}) =\frac{1}{N}\sum _{i=1}^{N}L(y_i,f(\mathbf {x_i};\textbf{w}))+\alpha R(\textbf{w}) \end{aligned}$$

where, $L(y_i,f(\mathbf {x_i};\textbf{w}))$ represents the loss function or error between the predictions of the model $f(\mathbf {x_i};\textbf{w})$ and the target values, $y_i$, of each sample $\mathbf {x_i}$ and $R(\textbf{w})$ is the regularization term. At each iteration, SGD minimizes this objective function using the derivative of the loss function for each parameter of the model:

2 $$\begin{aligned} \textbf{w}:=\textbf{w}-\eta \frac{\partial Q(\textbf{w})}{\partial \textbf{w}} \end{aligned}$$

where, $\eta$ is the learning rate.

The hinge loss is equivalent to the SVM classification:

3 $$\begin{aligned} L(y_i, f(\mathbf {x_i}, \textbf{w}))=max(0, 1-y_if(\mathbf {x_i};\textbf{w})), \end{aligned}$$

and the log loss corresponds to the logistic regression:

4 $$\begin{aligned} L(y_i, f(\mathbf {x_i}, \textbf{w}))=log(1+exp(-y_if(\mathbf {x_i};\textbf{w})). \end{aligned}$$

In addition to these two loss functions, we also incorporated three different regularization functions: L1 (eqn. 5), L2 (eqn. 6) norms, and Elastic Net (eqn. 7)^39^:

5 $$\begin{aligned} R_{L1}(\textbf{w})&= \sum _{j=1}^{m}|w_j| \end{aligned}$$

6 $$\begin{aligned} R_{L2}(\textbf{w})&= \frac{1}{2}\sum _{j=1}^{m}w_j^2 \end{aligned}$$

7 $$\begin{aligned} R_{elc}(\textbf{w})&= \frac{\rho }{2}\sum _{j=1}^{m}w_j^2 + (1-\rho )\sum _{j=1}^{m}|w_j|. \end{aligned}$$

Apart from these configurations, all of the hyperparameters were set to their default values.

### Preprocessing

The cell populations with less than 10 cells across the entire dataset were eliminated and then were log-normalized using $log_e(count+1)$. Each dataset was divided into batches using stratified k-fold sampling akin to^13^. One of these batches was left out as the test batch, and the remaining batches were fed to the classifiers sequentially. This process is the same as stratified K-fold cross-validation; however, rather than providing all the batches together except the test batch, the batches were provided individually for continual training of the classifiers.

**Table 1:** Overview of the datasets used for this study.

	Number of cells	Number of genes	Number of cell populations
Brain			
Allen Mouse Brain (AMB)	12,832	42,625	92
Human Lung			
CellBench 10X	3803	11,778	5
CellBench CEL-Seq2	570	12,627	5
Human Lung Cell Atlas (HLCA)	61,603	2000	23
Pancreas			
Baron Human	8569	17,499	13
Baron Mouse	1886	14,861	9
Muraro	2122	18,915	8
Segerstolpe	2133	22,757	9
Xin	1449	33,889	4
Whole Mus Musculus			
Tabula Muris (TM)	54,865	19,791	55
PBMC			
Zheng68K	65,943	20,387	11
Zheng-sorted	20,000	21,952	10
EQTL	24,439	22,229	11
*10Xv2	6444	33,694	9(4)
*10Xv3	3222	33,694	8(4)
*CEL-Seq	253	33,694	7(4)
*Drop-Seq	3222	33,694	9(4)
*inDrop	3222	33,694	7(4)
*Smart-Seq2	253	33,694	6(4)
*Seq-Well	3176	33,694	10(4)

*For inter-dataset experiments, different datasets were extracted such that they share the same type of cell populations (class labels).

### Datasets

In this study, we used a total of 20 datasets to evaluate the performance of various machine learning models for single-cell RNA sequencing (scRNA-seq) analysis in a CL scenario. The majority of the datasets utilized in the experiments came from the benchmark dataset of^13^. Brief information about the datasets is provided in Table 1. We expanded the benchmark more in order to represent different datasets with varying numbers of cells and genes and to evaluate where the CL strategy comes in handy.

For the intra-dataset experiments, we used 13 datasets, which all vary in the sequencing protocol used. We utilized all the datasets that were used for the intra-dataset experiments in^13^. They include the Allen Mouse Brain (AMB) dataset; 5 pancreatic datasets (Baron Mouse, Baron Human, Muraro, Segerstolpe, and Xin) from both mouse and human pancreatic cells; two CellBench datasets (10X and CEL-Seq2) from a mixture of five human lung cancer cell lines; the Tabula Muris (TM) dataset from whole Mus musculus; and two PBMC datasets (Zheng68K and Zheng Sorted). Zheng68K, Zheng sorted, and AMB92 (AMB dataset containing 92 cell populations) are described as the most complex datasets in^13^. We also included two datasets in our analysis: the PBMC-eQTL dataset, previously utilized in the hierarchical analysis by^14^, and a subset of the Human Lung Cell Atlas (HLCA) dataset described in^15^, which was updated from the original HLCA dataset in^34^. The subset comprises 61603 samples and 2000 highly expressed genes. The PBMC-eQTL dataset was sequenced using $10\times$ Chromium and consists of 24439 cells, 22229 genes, and eleven different cell populations^40^. The HLCA dataset is annotated with different annotation levels; the third level, consisting of 24 cell populations, was used in this study.

We also use the HLCA dataset to evaluate the performance of the classification models on the dataset that is projected in latent space. scANVI embeddings created by Michielsen et al. in their work^34^ were used in this experiment.

For the inter-dataset experiments, we utilized PbmcBench datasets, which are sequenced using 7 different protocols (10Xv2, Smart-Seq2, 10Xv3, CEL-Seq, Drop-Seq, inDrop, Seq-Well) and also used for the inter-dataset experiments in^13^. We used only pbmc1 datasets to evaluate the classification performance in a CL scenario in which each dataset from 7 protocols is used as the test set and fed the model batch by batch.

### Supplementary Information

Supplementary Information.

## Data Availability

All of the datasets analyzed in the current study are publicly available and can be downloaded from these Zenodo repositories: Intra and Inter Datasets^13^: https://doi.org/10.5281/zenodo.3357167 PBMC-eQTL^14^: https://zenodo.org/record/3736493#.ZGZgXHZBxPY HLCA latent space^15^: https://zenodo.org/record/6337966#.YqmGIidBx3g.

## Code availability

The codes used in this study are available in the Github repository, at https://github.com/gsaygili/CL4scRNA.
