# Supplementary material for: Continual learning approaches for single cell RNA sequencing data
Source: Sci Rep. 2023 Sep 15;13:15286. doi: 10.1038/s41598-023-42482-7 (PMC10504339; doi:10.1038/s41598-023-42482-7)
Supplement: Supplementary file 1 — Supplementary Information. [file 41598_2023_42482_MOESM1_ESM.pdf]

Supplementary Materials

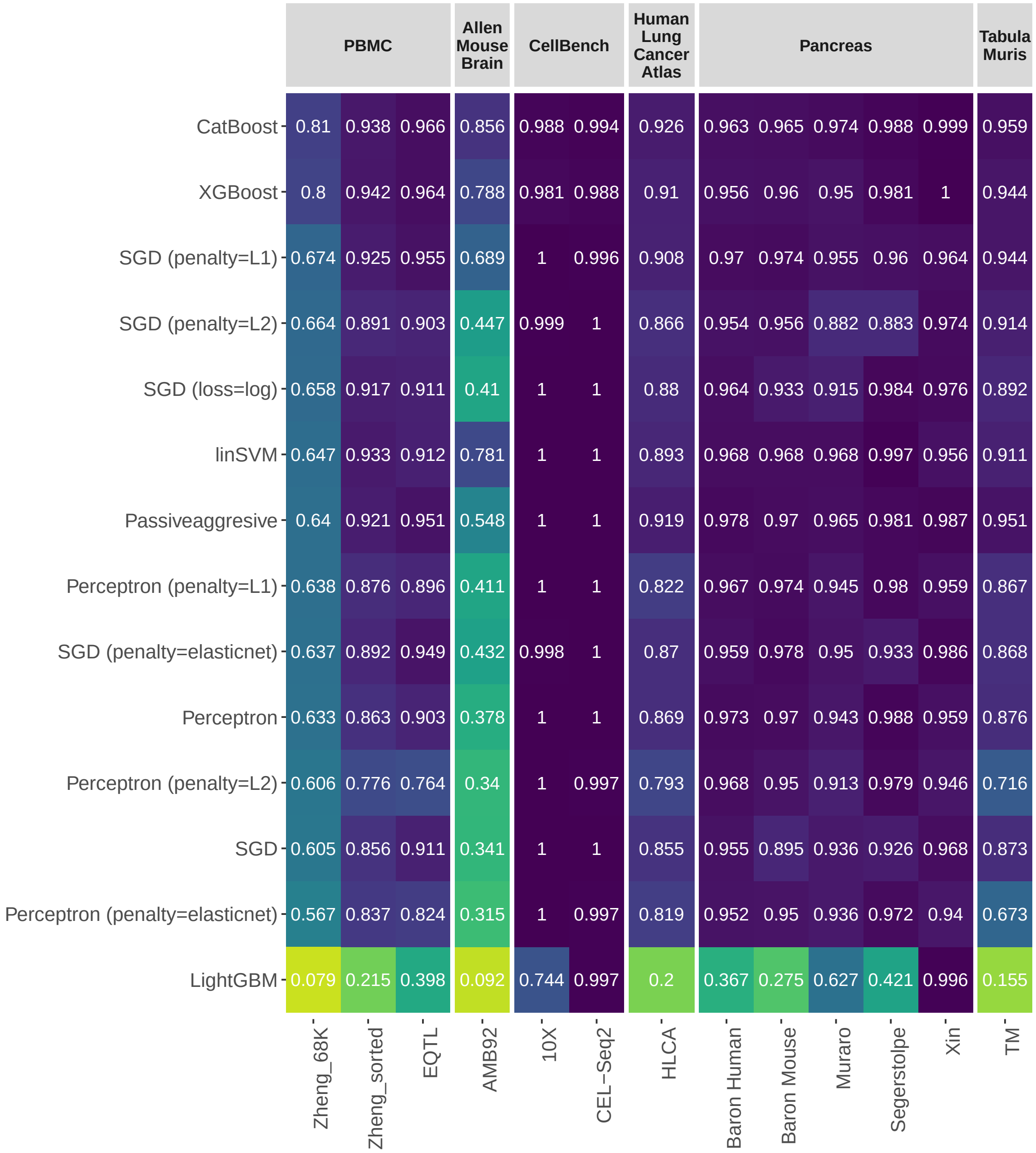

Figure 1. The intra-dataset experiment results that are sorted based on median F1-scores of challenging Zheng68K dataset for each classifier and for different penalty terms of the SGD, Perceptron classifiers.

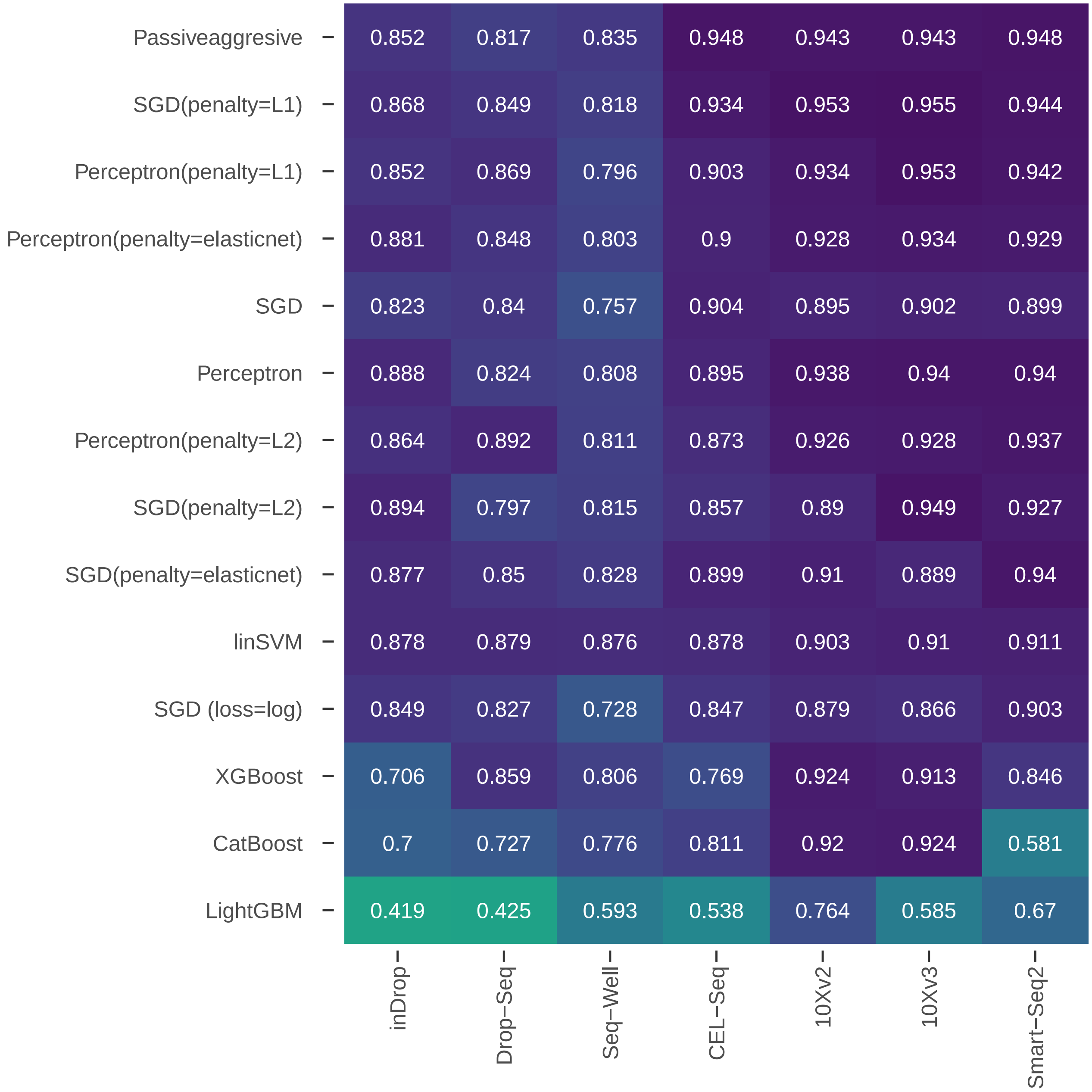

Figure 2. The inter-dataset experiment results that are sorted based on the mean of median F1-scores over all datasets for each classifier and for different penalty terms of the SGD, Perceptron classifiers.

	5 Batches	10 Batches	20 Batches
CatBoost	0.811	0.788	0.746
XGBoost	0.8	0.736	0.657
SGD(penalty=L1)	0.675	0.683	0.678
SGD(penalty=L2)	0.665	0.632	0.602
SGD (loss=log)	0.659	0.648	0.619
linSVM	0.647	0.634	0.627
Passiveaggresive	0.64	0.691	0.653
Perceptron(penalty=L1)	0.639	0.585	0.651
SGD(penalty=elasticnet)	0.637	0.614	0.668
Perceptron	0.633	0.628	0.532
Perceptron(penalty=L2)	0.607	0.413	0.474
SGD	0.605	0.649	0.645
Perceptron(penalty=elasticnet)	0.568	0.46	0.485
LightGBM	0.08	0.235	0.149

Figure 3. Median F1 scores for the comparison of the different batch sizes on Zheng68K dataset for each classifier and for different penalty terms of the SGD, Perceptron classifiers.
